# Risk-Association of *CYP11A1* Polymorphisms and Breast Cancer Among Han Chinese Women in Southern China

**DOI:** 10.3390/ijms13044896

**Published:** 2012-04-18

**Authors:** Minying Sun, Xuexi Yang, Changsheng Ye, Weiwen Xu, Guangyu Yao, Jun Chen, Ming Li

**Affiliations:** 1 School of Biotechnology, Southern Medical University, Guangzhou 510515, China; E-Mails: sunmy1220@163.com (M.S.); yxxzb@sohu.com (X.Y.); xu_sandy2006@126.com (W.X.); 2 Breast Center, Nanfang hospital, Southern Medical University, Guangzhou 510515, China; E-Mails: Yechsh2006@126.com (C.Y.); ygy531@163.com (G.Y.); chenjunlqq@163.com (J.C.); 3 Da An Gene Co., Ltd. of Sun Yat-sen University, Guangzhou 510665, China

**Keywords:** *CYP11A1*, breast cancer, single nucleotide polymorphism (SNP), susceptibility

## Abstract

Exposure to endogenous sex hormones has been reported as a risk factor for breast cancer. The *CYP11A1* gene encodes the key enzyme that catalyzes the initial and rate-limiting step in steroid hormone synthesis. In this study, the associations between single nucleotide polymorphisms (SNPs) in *CYP11A1* and breast cancer susceptibility were examined. Six SNPs in *CYP11A1* were genotyped using the MassARRAY IPLEX platform in 530 breast cancer patients and 546 healthy controls. Association analyses based on a χ^2^ test and binary logistic regression were performed to determine the odds ratio (*OR*) and 95% confidence interval (95% *CI*) for each SNP. Two loci (rs2959008 and rs2279357) showed evidence of associations with breast cancer risk. The variant genotype C/T-C/C of rs2959008 was significantly associated with a decreased risk (age-adjusted *OR*, 0.75; 95% *CI*, 0.58–0.96; *P* = 0.023) compared with the wild-type TT. However, the homozygous TT variant of rs2279357 exhibited increased susceptibility to breast cancer (age-adjusted *OR*, 1.44; 95% *CI*, 1.05–1.98; *P* = 0.022). The locus rs2959003 also showed an appreciable effect, but no associations were observed for three other SNPs. Our results suggest that polymorphisms of *CYP11A1* are related to breast cancer susceptibility in Han Chinese women of South China.

## 1. Introduction

Breast cancer is one of the most common malignancies among women worldwide and the incidence rate is increasing. Compared with Western countries, the peak age of breast cancer incidence is much earlier and the mortality rate is increasing in Asian populations [1]. Breast cancer is a heterogeneous disease caused by multiple genetic and environmental factors. Many candidate genes that may cause breast cancer have been identified in the past few years including high penetrance genes (*BRCA1/2*, *TP53* and *PTEN*), moderate penetrance genes (*ATM*, *CHEK2*, *PALB2* and *BRIP*), and low penetrance genes (*FGFR2*, *ESR1* and *TOX3*) [2–7]. Inherited mutations among those genes predispose to high risks of breast cancer. Life time risks of breast cancer among *BRCA1/2* mutation carriers are 82% [8]. Mutations of *CHEK2*, *PALB2*, and *BRIP1* have a 2.34, 2.3, and 2.0 fold increase risk for breast cancer compared to the normal population, respectively [5–7]. Numerous genome-wide association studies (GWAS) also have identified approximately fifty common genetic loci for breast cancer risk in low penetrance genes such as *FGFR2*, *ESR1*, *LSP1*, *MAP3K1*, *RAD51L1* and *TOX3* [9–13]. Although each locus confers no more than 1.4 odds ratio, a combination of these variants may act cumulatively to increase breast cancer risk. As more breast cancer susceptibility genes of different penetrances are identified, more appropriate genetic tests and risk reduction strategies can be developed.

Previous reports indicate that prolonged exposure to endogenous sex hormones, especially estrogens and progestogens, can increase the risk of breast cancer [14,15]. High levels of endogenous sex hormones are considered crucial factors associated with breast cancer susceptibility. Several case-control studies have also shown evidences that polymorphisms in steroid hormone biosynthesis genes are associated with breast cancer susceptibility [16–19]. The *CYP11A1* (cytochrome P-450 11A1) gene is located at 15q23-q24 and consists of nine exons spanning a total of 29,864 bp. This gene encodes the cholesterol side chain cleavage enzyme P450scc, a member of the cytochrome P450 superfamily of enzymes, which resides on the mitochondrial inner membrane and catalyzes the conversion of cholesterol to pregnenolone, the initial and rate-limiting step in steroid hormone synthesis [20]. *CYP11A1* is primarily expressed in steroidogenic tissues, such as the adrenal cortex, gonads, and placenta. Although P450scc is always active, genetic variants of *CYP11A1* may alter its expression and activities, and thus result in certain hormone-related diseases. Polymorphisms of *CYP11A1* have been detected as potential markers in different hormone-dependent diseases, including breast cancer [16–19], polycystic ovary syndrome (PCOS) [21], prostate cancer [22,23], and endometrial cancer [24]. Genetic architecture is different among populations and the data reported to date concentrate mainly on Western populations or pentanucleotide [(TAAAA)_n_] repeat polymorphisms. Here, we investigated the associations between single nucleotide polymorphisms (SNPs) in *CYP11A1* and breast cancer among Han Chinese women in Guangdong province, South China. Six SNPs in *CYP11A1* (rs2959008, rs2959003, rs2279357, rs11638442, rs2073475, and rs16968478) were genotyped to perform a case-control study in women from Guangdong province.

## 2. Results and Discussion

### 2.1. Study Subjects

All subjects included in this study were Han Chinese women. The mean ages of the patients and controls were 48.48 ± 10.18 and 44.59 ± 11.26 years, respectively. An independent-sample *t*-test indicated a significant difference in age distribution between the two groups (*P* < 0.05, data not shown), so all statistical analyses were subsequently adjusted by age.

### 2.2. Hardy-Weinberg Equilibrium (HWE), Linkage Disequilibrium (LD) and Haplotype Analysis

**Figure 1:**
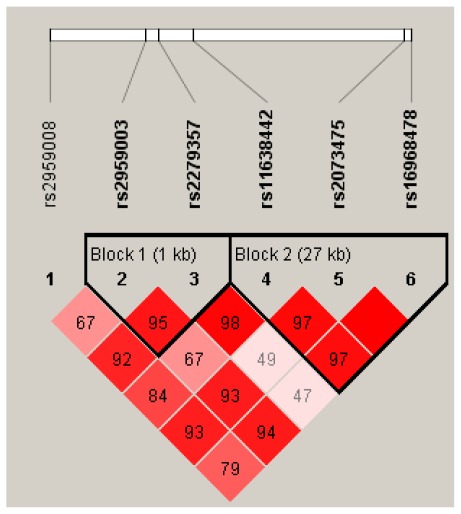
Linkage disequilibrium (LD) pattern among six SNPs by Haploview analysis. Numbers inside the boxes represent D′ values for LD, and the miss number is 100.

All SNPs conformed to Hardy-Weinberg equilibrium (HWE) among both cases and controls (*P* > 0.05) with minor allele frequency (MAF) ≥ 0.22 (Table 1). The SNPs rs11638442, rs2073475, and rs16968478 were in high or complete linkage disequilibrium as well as rs2959003 and rs2279357 (supplementary material). D′ values are shown in Figure 1. The SNP rs2959008 had a low LD compared to the others.

Six main haplotypes of *CYP11A1* were considered in the analysis for all subjects. The results of individual haplotype analysis and breast cancer are shown in Table 2. The haplotype TTTCAG was most common in both case and control groups. The distribution frequency of haplotype CCCCGA was significantly different between the two groups and protective against the development of breast cancer (*OR*, 0.64; 95% *CI*, 0.48–0.86; *P* = 0.0033).

### 2.3. Polymorphisms of CYP11A1 and Breast Cancer Risk

The genotype distributions of rs2959008 and rs2279357 were significantly different between cases and controls in the chosen genetic model (Table 3). For rs2959008, the variant genotype C/T-C/C was significantly associated with a decreased risk of breast cancer (*OR*, 0.75; 95% *CI*, 0.58–0.96; *P* = 0.023) compared with the wild-type TT. Individuals carrying the C allele showed protection from breast cancer (*OR*, 0.80; 95% *CI*, 0.67–0.96; *P* = 0.018). In contrast to rs2959008, the homozygous TT variant and allele T of rs2279357 were associated with elevated susceptibility to breast cancer (*OR*, 1.44; 95% *CI*, 1.05–1.98; *P* = 0.022 and *OR*, 1.23; 95% *CI*, 1.04–1.47; *P* = 0.018, respectively). A correlation between the rs2959003 polymorphism and breast cancer was also observed under the log-additive model. Three other SNPs (rs11638442, rs2073475, and rs16968478) did not show significant differences between the groups in the present study (supplementary material).

A further association analysis was conducted to identify the interactions of two susceptibility-associated SNPs, rs2959008 and rs2279357, and their impact on the risk of breast cancer. The results indicated that the united genotype TT-TT of rs2959008 and rs2279357 showed increased breast cancer risk (*OR*, 1.65; 95% *CI*, 1.18–2.31; *P* = 0.003) (Table 4). There was also a marginal association between genotype CT/CC-TT and reduced breast cancer risk (*OR*, 0.12; 95% *CI*, 0.02–0.94; *P* = 0.044).

Breast cancer risk is partially determined by endogenous hormone levels [25]. Given the important role of P450scc in steroid sex hormone biosynthesis, this case-control study was performed among Han Chinese women in Guangdong province. All six SNPs (rs2959008, rs2959003, rs2279357, rs11638442, rs2073475 and rs16968478) are located in noncoding regions of *CYP11A1*, with the first four in introns and the latter two upstream of the gene. The results showed that rs2959008 and rs2279357 of the *CYP11A1* gene were strongly associated with breast cancer risk, as well as the interaction of the two SNPs.

Numerous studies have examined *CYP11A1* gene polymorphisms. The best studied region is the pentanucleotide [TTTTA]_n_ repeat (D15S520) located 528 bp upstream of the translational initiation site, which showed six common polymorphisms with four, six, seven, eight, nine, or ten [TTTTA] repeats. In the Chinese population, the main variants are the four-, six-, and eight-repeat alleles, which are correlated with risks of breast cancer [16,17] and polycystic ovarian syndrome [21]. The [TTTTA]_4_ and [TTTTA]_6_ alleles were also shown to be associated with prostate cancer in other ethnic groups [22,23]. However, the [(TAAAA)_n_] polymorphism was different from common biallelic variations in the present study.

Setiawan *et al*. systematically evaluated genetic variations spanning 67 kb of *CYP11A* and suggested that common variations in *CYP11A* may be related to breast cancer susceptibility in a western Multiethnic Cohort Study, but no particular SNP or haplotype was identified as the most likely causal variant [18]. Mammographic density has also been shown to be a breast cancer risk factor, and is strongly associated with hormone exposure profile [26]. In Swedish women, our five tag SNPs (rs2959008, rs2959003, rs2279357, rs11638442, and rs16968478) were significantly related to mammographic density in single SNP analysis, but the relationship disappeared after correction for multiple testing [27]. Another three tag SNPs (rs4555110, rs3825944, and rs7173655) were significantly associated with increased (1.3–1.4-fold) endometrial cancer risk in women in Boston, USA [24]. Along with another 11 loci, polymorphisms of *CYP11A1* were also confirmed to be associated with hypertension and blood pressure (BP) in the Japanese population, with the presence of more of these alleles indicating higher risk [28]. These results were mainly based on Western populations. Haplotype analysis indicated that the promoter haplotype of *CYP11A1* was associated with increased risk of breast cancer among Chinese women in Shanghai, but three of our SNPs (rs2959008, rs2959003, and rs16968478) were not included [19]. Here, we focused on the Han Chinese population in Guangdong province and identified breast cancer-related SNPs. Six SNPs that have seldom been reported in the Han Chinese population were selected to verify the association. The results suggested that the haplotype CCCCGA had a protective effect against breast cancer. Two single SNPs, rs2959008 and rs2279357, were significantly associated with breast cancer risk and interaction analysis also demonstrated that the united genotype TT-TT of these two SNPs showed much higher risk of breast cancer.

## 3. Experimental Section

### 3.1. Subjects

The subjects participated in our study were recruited between April 2009 and October 2011 from Nanfang Hospital, Southern Medical University, Guangzhou, Guangdong Province, China. All participants were permanent residents of Guangdong from the Han Chinese population. Clinical information for each subject was collected from medical records.

#### 3.1.1. Patients of Breast Cancer

A total of 621 female patients with breast cancer were recruited after confirmed by pathological diagnosis through breast clinics. 550 (88.6%) of them were approached with their written informed consent and blood samples were collected. The age ranged from 22 to 80 years old. Other 71 patients refused to provide blood samples. Blood samples from 14 patients were unusable for DNA extraction due to transportation and storage at −70 °C After genotyping, 6 samples were excluded as all six SNPs could not be determined. Therefore, 530 case subjects were included in the final statistical analysis.

#### 3.1.2. Controls

550 healthy control women were randomly selected from outpatients ranging in age from 16 to 84 years old during the same period with no history of cancer or other breast-related diseases as determined by molybdenum target mammography and color Doppler ultrasonography and blood samples were collected. However, 4 samples failed in genotyping and 546 healthy controls were included in the final statistical analysis.

The study protocol was approved by the Clinical Research Ethics Committee of Nanfang Hospital and written informed consent was obtained from all participants.

### 3.2. DNA Extraction

Peripheral blood samples were collected from the participants and stored at −70 °C until DNA extraction. Genomic DNA was extracted from peripheral blood samples using an E.Z.N.A.™ blood DNA kit (Omega Bio-Tek, Norcross, GA, USA) according to the manufacturer’s protocol.

### 3.3. Genotyping

For each SNP, a pair of amplification primers and an extension primer was designed using Assay Design 3.1 software (Sequenom, San Diego, CA, USA). Genotypes were generated using the SEQUENOM MassARRAY matrix-assisted laser desorption/ionization time-of-flight (MALDI-TOF) mass spectrometry platform (Sequenom, San Diego, CA, USA) according to the manufacturer’s instructions. This is one of the most powerful tools for detecting insertions, deletions, substitutions, and other polymorphisms in amplified DNA, and allows rapid, efficient, and high-throughput detection without any interactions. The overall call rates ranged from 99.63% to 100%.

### 3.4. Statistical Analysis

Hardy–Weinberg equilibrium (HWE) and linkage disequilibrium (LD) for the six SNPs were calculated using Haploview 4.2 (Daly Lab, Cambridge, MA, USA). The genotype and allele distributions as well as the interactions of SNPs between case and control subjects were compared. Multiple inheritance models (codominant, dominant, recessive, overdominant, and log-additive) were chosen to evaluate the associations between each SNP and breast cancer risk. The odds ratio (*OR*) and 95% confidence interval (95% *CI*) were evaluated by binary logistic regression analyses. There was a significant difference in age distribution between case and control groups as described previously, so the age was adjusted by setting as a covariate in association analyses for each SNP. Statistical analyses were implemented using SPSS 13.0 software (SPSS, Chicago, IL, USA) and the Web-based tool SNPstats [29]. All comparisons were two-sided and *P* < 0.05 was regarded as statistically significant.

## 4. Conclusions

In summary, our results showed that rs2959008 and rs2279357 of the *CYP11A1* gene were significantly associated with breast cancer susceptibility among Guangdong Han Chinese women, and the interaction of these two SNPs was associated with elevated risk. The haplotype CCCCGA of our six SNPs also had a protective effect against the development of breast cancer. The biological mechanisms behind these associations remain unknown. Therefore, further comprehensive investigations of steroid hormone biosynthesis and metabolism gene variations combined with other risk factors are required to identify biomarkers for inherited breast cancer susceptibility.

## Supplementary Material



## Figures and Tables

**Table 1:** Genotype characteristics of the six single nucleotide polymorphisms (SNPs).

SNP	Alleles	MAF^a^	HWE^b^ (*P*-value)	

			Case	Control
rs2959008	C/T	0.36/C	0.976	0.587
rs2959003	C/T	0.38/C	0.852	0.519
rs2279357	C/T	0.43/T	0.403	0.803
rs11638442	C/G	0.22/G	0.659	0.674
rs2073475	A/G	0.44/A	0.951	0.765
rs16968478	A/G	0.46/G	0.834	0.732

a Minor Allele Frequency;

b Hardy-Weinberg equilibrium.

**Table 2:** Association between haplotypes and breast cancer for six SNPs in *CYP11A1*.

Haplotype^a^	Frequencies			*OR* (95% *CI*)^b^	*P-*value

	Total	Control	Case		
TTTCAG	0.2936	0.2755	0.314	1	-
CCCGGA	0.153	0.1567	0.1495	0.82 (0.62–1.08)	0.15
CCCCGA	0.1309	0.1495	0.1111	**0.64 (0.48–0.86)**	0.0033
TTCCAG	0.1273	0.1333	0.1203	0.78 (0.58–1.06)	0.11
TTTCGA	0.1177	0.1079	0.1269	1.03 (0.76–1.40)	0.83
TCCCGA	0.0673	0.071	0.0638	0.83 (0.57–1.20)	0.32
rare	0.1102	0.1061	0.1145	0.92 (0.67–1.28)	0.64

a Haplotypes were constructed for rs2959008, rs2959003, rs2279357, rs11638442, rs2073475 and rs16968478;

b *OR* (95% *CI*) was adjusted for age and the bold values indicate *P* < 0.05.

**Table 3:** The genotype distributions (%) and association of breast cancer risk.

SNP	Model	Genotype	Control	Case	*OR* (95% *CI*) a	*P*-value
rs2959008	Codominant	T/T	205 (37.5%)	229 (43.2%)	1	0.057
		C/T	264 (48.4%)	239 (45.1%)	0.77 (0.59–1.00)	
		C/C	77 (14.1%)	62 (11.7%)	**0.67 (0.45–0.99)**	

	Dominant	T/T	205 (37.5%)	229 (43.2%)	1	0.023
		C/T-C/C	341 (62.5%)	301 (56.8%)	**0.75 (0.58–0.96)**	

	Recessive	T/T-C/T	469 (85.9%)	468 (88.3%)	1	0.16
		C/C	77 (14.1%)	62 (11.7%)	0.77 (0.53–1.11)	

	Overdominant	T/T-C/C	282 (51.6%)	291 (54.9%)	1	0.2
		C/T	264 (48.4%)	239 (45.1%)	0.85 (0.67–1.09)	

	Log-additive	-	-	-	**0.80 (0.67–0.96)**	0.018

rs2959003	Codominant	T/T	196 (36%)	218 (41.3%)	1	0.092
		C/T	255 (46.9%)	241 (45.6%)	0.84 (0.64–1.10)	
		C/C	93 (17.1%)	69 (13.1%)	**0.67 (0.46–0.97)**	

	Dominant	T/T	196 (36%)	218 (41.3%)	1	0.072
		C/T-C/C	348 (64%)	310 (58.7%)	0.79 (0.62–1.02)	

	Recessive	T/T-C/T	451 (82.9%)	459 (86.9%)	1	0.078
		C/C	93 (17.1%)	69 (13.1%)	0.74 (0.52–1.04)	

	Overdominant	T/T-C/C	289 (53.1%)	287 (54.4%)	1	0.62
		C/T	255 (46.9%)	241 (45.6%)	0.94 (0.74–1.20)	

	Log-additive	-	-	-	**0.82 (0.69–0.98)**	0.03

rs2279357	Codominant	C/C	193 (35.4%)	164 (30.9%)	1	0.048
		C/T	265 (48.6%)	253 (47.7%)	1.14 (0.87–1.50)	
		T/T	87 (16%)	113 (21.3%)	**1.56 (1.09–2.22)**	

	Dominant	C/C	193 (35.4%)	164 (30.9%)	1	0.1
		C/T-T/T	352 (64.6%)	366 (69.1%)	1.24 (0.96–1.61)	

	Recessive	C/C-C/T	458 (84%)	417 (78.7%)	1	0.022
		T/T	87 (16%)	113 (21.3%)	**1.44 (1.05–1.98)**	

	Overdominant	C/C-T/T	280 (51.4%)	277 (52.3%)	1	0.82
		C/T	265 (48.6%)	253 (47.7%)	0.97 (0.76–1.24)	

	Log-additive	-	-	-	**1.23 (1.04–1.47)**	0.018

a *OR* (95% *CI*) was adjusted for age and the bold values indicate *P* < 0.05.

**Table 4:** Interaction for rs2959008 and rs2279357 between case and control group.

Genotype	Control	Case	χ^2^	*P*-value	*OR* (95% *CI*)^a^	*P*-value
TT-TT	77 (14.1%)	112 (21.1%)	15.57	0.001	**1.65 (1.18–2.31)**	0.003
TT-CT/CC	127 (23.3%)	117 (22.1%)			1.10 (0.81–1.48)	0.551
CT/CC-TT	10 (1.8%)	1 (0.2%)			**0.12 (0.02–0.94)**	0.044
CT/CC-CT/CC	331 (60.7%)	300 (56.6%)			1	-

a *OR* (95% *CI*) was adjusted for age and the bold values indicate *P* < 0.05.
