# Supplementary material for: Risk-Association of CYP11A1 Polymorphisms and Breast Cancer Among Han Chinese Women in Southern China
Source: Int J Mol Sci. 2012 Apr 18;13(4):4896–905. doi: 10.3390/ijms13044896 (PMC3344254; doi:10.3390/ijms13044896)
Supplement: Supplementary file 1 [file ijms-13-04896-s001.pdf]

Supplementary Material

Table S1. The genotype distributions (%) and association of breast cancer risk for rs11638442, rs2073475 and rs16968478.

SNP	Model	Genotype	Control	Case	*OR* (95% *CI*)[a]	*P*-value
rs11638442	Codominant	C/C	337 (61.7%)	316 (59.7%)	1	0.9
		C/G	186 (34.1%)	188 (35.5%)	1.04 (0.80-1.34)	
		G/G	23 (4.2%)	25 (4.7%)	1.13 (0.62-2.05)	
	Dominant	C/C	337 (61.7%)	316 (59.7%)	1	0.72
		C/G-G/G	209 (38.3%)	213 (40.3%)	1.05 (0.82-1.34)	
	Recessive	C/C-C/G	523 (95.8%)	504 (95.3%)	1	0.72
		G/G	23 (4.2%)	25 (4.7%)	1.12 (0.62-2.01)	
	Overdominant	C/C-G/G	360 (65.9%)	341 (64.5%)	1	0.84
		C/G	186 (34.1%)	188 (35.5%)	1.03 (0.80-1.33)	
	Log-additive	---	---	---	1.05 (0.85-1.29)	0.67
rs2073475	Codominant	G/G	180 (33%)	162 (30.7%)	1	0.75
		G/A	264 (48.4%)	261 (49.5%)	1.08 (0.82-1.43)	
		A/A	102 (18.7%)	104 (19.7%)	1.14 (0.80-1.62)	
	Dominant	G/G	180 (33%)	162 (30.7%)	1	0.49
		G/A-A/A	366 (67%)	365 (69.3%)	1.10 (0.84-1.42)	
	Recessive	G/G-G/A	444 (81.3%)	423 (80.3%)	1	0.6
		A/A	102 (18.7%)	104 (19.7%)	1.09 (0.80-1.48)	
	Overdominant	G/G-A/A	282 (51.6%)	266 (50.5%)	1	0.82
		G/A	264 (48.4%)	261 (49.5%)	1.03 (0.81-1.31)	
	Log-additive	---	---	---	1.07 (0.90-1.27)	0.45
rs16968478	Codominant	A/A	163 (29.9%)	147 (27.8%)	1	0.81
		G/A	267 (48.9%)	266 (50.3%)	1.08 (0.81-1.44)	
		G/G	116 (21.2%)	116 (21.9%)	1.11 (0.79-1.57)	
	Dominant	A/A	163 (29.9%)	147 (27.8%)	1	0.53
		G/A-G/G	383 (70.2%)	382 (72.2%)	1.09 (0.83-1.43)	
	Recessive	A/A-G/A	430 (78.8%)	413 (78.1%)	1	0.71
		G/G	116 (21.2%)	116 (21.9%)	1.06 (0.79-1.42)	
	Overdominant	A/A-G/G	279 (51.1%)	263 (49.7%)	1	0.79
		G/A	267 (48.9%)	266 (50.3%)	1.03 (0.81-1.32)	
	Log-additive	---	---	---	1.06 (0.89-1.25)	0.53

a: *OR* (95% *CI*) was adjusted for age.

Table S2. The results of Hardy-Weinberg equilibrium (HWE) test for all SNPs.

SNP	Genotype	Control	*P*-value of HWE	Case	*P*-value of HWE
rs2959008	T/T	205 (37.5%)		229 (43.2%)	
	C/T	264 (48.4%)	0.587	239 (45.1%)	0.976
	C/C	77 (14.1%)		62 (11.7%)	
rs2959003	T/T	196 (36%)		218 (41.3%)	
	C/T	255 (46.9%)	0.519	241 (45.6%)	0.852
	C/C	93 (17.1%)		69 (13.1%)	
rs2279357	C/C	193 (35.4%)		164 (30.9%)	
	C/T	265 (48.6%)	0.803	253 (47.7%)	0.403
	T/T	87 (16%)		113 (21.3%)	
rs11638442	C/C	337 (61.7%)		316 (59.7%)	
	C/G	186 (34.1%)	0.674	188 (35.5%)	0.660
	G/G	23 (4.2%)		25 (4.7%)	
rs2073475	G/G	180 (33%)		162 (30.7%)	
	G/A	264 (48.4%)	0.765	261 (49.5%)	0.951
	A/A	102 (18.7%)		104 (19.7%)	
rs16968478	A/A	163 (29.9%)		147 (27.8%)	
	G/A	267 (48.9%)	0.732	266 (50.3%)	0.834
	G/G	116 (21.2%)		116 (21.9%)	

© 2012 by the authors; licensee MDPI, Basel, Switzerland. This article is an open access article distributed under the terms and conditions of the Creative Commons Attribution license (http://creativecommons.org/licenses/by/3.0/).
